# Mitochondrial NHE1: a newly identified target to prevent heart disease

**DOI:** 10.3389/fphys.2013.00152

**Published:** 2013-06-28

**Authors:** Bernardo V. Alvarez, María C. Villa-Abrille

**Affiliations:** Centro de Investigaciones Cardiovasculares, Consejo Nacional de Investigaciones Científicas y Técnicas Facultad de Ciencias Médicas, Universidad Nacional de La PlataLa Plata, Argentina

**Keywords:** ischemia, mitochondrial permeability transition pore, NHE1, mitochondrial swelling, siRNA

## Abstract

Mitochondrial damage has been associated with early steps of cardiac dysfunction in heart subjected to ischemic stress, oxidative stress and hypertrophy. A common feature for the mitochondrial deterioration is the loss of the mitochondrial membrane potential (ΔΨ m) with the concomitant irreversible opening of the mitochondrial permeability transition pore (MPTP) which follows the mitochondrial Ca^2+^ overload, and the subsequent mitochondrial swelling. We have recently characterized the expression of the Na^+^/H^+^ exchanger 1 (mNHE1) in mitochondrial membranes. This surprising observation provided a unique target for the prevention of the Ca^2+^-induced MPTP opening, based on the inhibition of the NHE1 m. In this line, inhibition of NHE1 m activity and/or reduction of NHE1 m expression decreased the Ca^2+^-induced mitochondrial swelling and the release of reactive oxygen species (ROS) in isolated cardiac mitochondria and preserved the ΔΨ m in isolated cardiomyocytes. Mitochondrial NHE1 thus represents a novel target to prevent cardiac disease, opening new avenues for future research.

## Sodium/proton exchanger (NHE)

Sodium/proton exchangers (NHE) are a family of integral membrane proteins present in most organisms. These transporters which catalyze the electroneutral exchange of one intracellular H^+^ for one extracellular Na^+^ across membrane along their concentrations gradient are crucial for control of intracellular pH (pH_i_) and cell volume, and, cell migration and proliferation.

The first NHE isoform to be identified was NHE1, which has a ubiquitous tissue distribution in mammals (Sardet et al., 1989). Since its discovery, nine other human isoforms have been identified (NHE2–NHE10) (Fliegel, 2008; Lee et al., 2008). While NHE1–NHE6 reside in the plasma membrane or recycling endosomes, NHE7–NHE9 are located inside the cell rather than the plasma membrane (Fliegel, 2008). The NHE10 is expressed in the surface of osteoclast (Lee et al., 2008).

## NHE1 in the heart

NHE1 is the most studied isoform that accumulates preferentially in microdomains of cells membranes, concentrating along the basolateral membrane of epithelia (Biemesderfer et al., 1992) and the intercalated disks and t-tubules of cardiac myocytes (Petrecca et al., 1999). The sarcolemmal NHE1 is the major Na^+^ influx pathway found in the plasma membrane of cardiac cells.

NHE1 is an integral membrane glycoprotein with a predicted molecular mass of 85 kDa. NHE1 can be separated into an N-terminal, membrane-associated domain, and a long C-terminal tail, with both the N-and C-terminal domains being cytoplasmic (Wakabayashi et al., 1997), (Orlowski and Grinstein, 1997). NHE1 is expressed ubiquitously in mammalian cells, where it electroneutrally exchanges one intracellular H^+^ for one extracellular Na^+^, thus regulating pH_i_. The membrane domain composed of 12 transmembrane regions is associated with ionic transport (Wakabayashi et al., 1992), and contains the allosteric H^+^ sensor site that determines the exquisite sensitivity of the exchanger to intracellular H^+^. The cytoplasmic domain is involved in the regulation of the activity of the exchanger by several mechanisms. Removal of the distal region of the cytosolic tail causes a shift of the pH_i_ sensitivity to the acidic side and an important inhibition of NHE1 activation by growth factors (Fliegel and Karmazyn, 2004). Cytoplasmic tail contains several phosphorylation sites and a high affinity and a low-affinity calmodulin binding sites (Bertrand et al., 1994). The high-affinity binding site functions as an “autoinhibitory domain” that binds Ca^2+^-bound calmodulin and allows activation of the exchanger. Deletion of this domain constitutively activates NHE1 and mimics elevated intracellular [Ca^2+^] (Wakabayashi et al., 1994). In addition, the cytoplasmic tail contains a binding site for the calcineurin B homolog protein CHP1, an essential cofactor for NHE1 (Pang et al., 2001). The exchanger is phosphorylated by different protein kinases in response to hormone and growth factor stimulation, as well as sustained acidosis (Sardet et al., 1990, 1991; Haworth et al., 2003).

Under basal conditions, NHE1 exchanger is relatively quiescent and its activity relies only on the extrusion of the H^+^ produced by the metabolic activity of cells as well as the H^+^ that enters the cell through acid-loading mechanisms. However, the exchanger has an exquisite sensitivity to the increase in intracellular H^+^ levels and enhances its activity once pH_i_ drops below a threshold level by allosterically “sensing” pH_i_, and by being phosphorylated or by interacting with some associated proteins, thus promoting the rapid extrusion of acid (Leem et al., 1999). NHE1 is constitutively phosphorylated in resting cells, but further phosphorylation is induced by several stimuli acting through G-protein-coupled receptors such as α1-adrenergic receptors, angiotensin II (Ang II), and endothelin-1 (ET-1). Kinases such as the Ca^2+^-/calmodulin-dependent kinase II (Fliegel et al., 1992), the extracellular signal-regulated kinase (ERK) (Moor and Fliegel, 1999), 90 kDa ribosomal S6 kinase (p90 rsk) (Takahashi et al., 1999), p38 mitogen-activated kinase (p38 MAPK) (Khaled et al., 2001), p160 ROCK (Tominaga et al., 1998), and the Nck-interacting kinase (NIK) (Yan et al., 2001) putatively phosphorylate NHE1 to modulate NHE1 activity. In addition, both protein kinases C and D are thought to influence NHE1 activity in response to growth factor and hormone stimulation without a direct phosphorylation of the exchanger (Fliegel et al., 1992; Haworth et al., 1999). NHE1 is also susceptible to dephosphorylation by protein phosphatases such as PP1 (Misik et al., 2005) and PP2A (Snabaitis et al., 2006). Carbonic anhydrase II (CAII) binds to the regulatory cytosolic domain of NHE1 enhancing its activity. Phosphorylation of the C terminus of NHE1 greatly increased the binding of CAII. This binding was shown to involve a protein–protein interaction, suggesting that both proteins constitute a complex. The inhibition of CAII decreased NHE1 activity significantly (Li et al., 2002).

As previously described, the NHE1, relatively quiescent under basal conditions, however, becomes highly active during ischemia in response to intracellular acidosis, leading to NHE1-mediated Na^+^ entry into the cell (Karmazyn, 1999a; Karmazyn et al., 1999). Inhibition of [Na^+^]_i_ accumulation by increasing NHE activity and prevention of Ca^2+^ overload via Na^+^/Ca^2+^ exchanger have been proposed as potential mechanisms of cardioprotection by NHE1 blockade (Avkiran, 1999). NHE exchanger inhibitors have proven to protect the heart against ischemia/reperfusion (I/R) injury (Karmazyn, 1999a,b). Moreover, the positive effect that NHE1 inhibition exerts on left ventricular systolic function during revascularization therapy after acute myocardial infarction has been documented (Rupprecht et al., 2000). The protection of the ischemic myocardium by NHE1 inhibition after the onset of reperfusion has been described (Rohmann et al., 1995; Gumina et al., 1998; Hurtado and Pierce, 2000). The decrease in myocardial infarct size, through interference with the action of reactive oxygen species (ROS) generation at the beginning of reperfusion, has also been reported (Koerner et al., 1991; Tanaka et al., 1994; McDonald et al., 1999; Sahna et al., 2002). Fantinelli et al. (2006) described that pharmacological interventions, ROS scavenging and NHE1 inhibition, when applied together and at their maximal effective concentration, did not induce protection further than that obtained separately by each pharmacologic procedure. These findings suggest that both interventions act through a common pathway. The authors proposed that in addition to the effect of preventing intracellular Na+ increase, NHE1 inhibition by cariporide decreases ROS-induced damage.

NHE1 activity is controlled by pH_i_ and numerous other factors, such as hormones, catecholamines, enzymes, and mechanical stimuli, known to be associated with heart disease (Avkiran and Haworth, 2003; Cingolani et al., 2008; Villa-Abrille et al., 2010; De Giusti et al., 2011). Furthermore, cardiac expression of an activated form of NHE1 that lacks the calmodulin-binding inhibitory domain was sufficient by itself to initiate cardiac hypertrophy and heart failure (Nakamura et al., 2008). Constitutively, active NHE1 leads to pathological changes in activation of the Ca^2+^-dependent pro-hypertrophic signaling molecules, calcineurin and CaMKII (Nakamura et al., 2008). Similarly, in heart over-expression of activated NHE1 was recently found to elicit specific pathways of gene activation, inducing an increase in cross-sectional area of cardiomyocyte, interstitial fibrosis, and depressed cardiac function, in transgenic mice (Xue et al., 2010).

## NHE1 and mitochondria

Emerging evidence supports the fact that mitochondrial dysfunction underlies the causes of numerous cardiac diseases (for review, see Baines, 2010). The mitochondrial death pathway features the sequential loss of mitochondrial membrane potential (ΔΨ m), which is accompanied by opening of the mitochondrial permeability transition pore (MPTP), release of ROS and diverse toxic proteins which promote the activation of proteolytic activity of caspases (Teshima et al., 2003). When the MPTP opens, the permeability barrier of the inner membrane becomes disrupted and causes the free movement of protons across it, inducing uncoupling of the oxidative phosphorylation and mitochondria swelling. The MPTP is a large conductance pore thought to be activated by ROS, by increased mitochondrial Ca^2+^ levels and by dissipation of the mitochondrial ΔΨ m (Akao et al., 2003; Javadov and Karmazyn, 2007). The MPTP opening can be further increased when Ca^2+^ overload is accompanied by oxidative stress, adenine nucleotide depletion, and elevated phosphate concentrations. Moreover, a decrease in mitochondrial anion superoxide production induced by two known enhancers of ROS production, Ang II and ET-1, upon NHE1 inhibition has been reported (Garciarena et al., 2008). Furthermore, the increase in ROS production induced by the opening of the mitochondrial ATP-dependent K^+^ channel was abolished by cariporide (Garciarena et al., 2008).

Studies on the MPTP and its role in reperfusion injury and cardiac hypertrophy have proven fruitful (Piot et al., 2008; Halestrap and Pasdois, 2009). Duchen et al. (1993) and Griffiths and Halestrap (1993) showed that delayed opening of MPTP by the MPTP inhibitor, cyclosporine A (CsA), protects ischemic/reperfusion (I/R) injury in perfused rat hearts. Recently, Piot et al. (2008) reported a decrease in infarct size in patients with acute myocardial infarction by CsA administered at the time of reperfusion. Interestingly, a role of mitochondria in determining cardiac hypertrophy after myocardial infarction has also been proposed (Karmazyn, 1988; Caldiz et al., 2007; Javadov et al., 2008, 2009; Cingolani et al., 2010). NHE1 inhibition was one of the most promising therapeutic strategies for I/R injury based on experimental animal studies. However controversial results were obtained using NHE1 inhibitors in clinical trials: cariporide (GUARDIAN) (Theroux et al., 2000) or EXPEDITION (Mentzer et al., 2008) and Eniporide (ESCAMI) (Zeymer et al., 2001) (for review, see Murphy and Allen, 2009). These trials were unable to demonstrate a significant reduction in mortality when these compounds were tested in patients after acute myocardial infarction (Theroux et al., 2000; Zeymer et al., 2001) or were suspended early due to undesired effects (Mentzer et al., 2008). In a subgroup of patients who underwent coronary artery bypass graft surgery and were treated with cariporide, a 25% improvement in cardiac performance was detected. Similar beneficial effects were seen in a small trial of 100 patients who received cariporide before percutaneous coronary angioplasty (Rupprecht et al., 2000).

**Figure 1:**
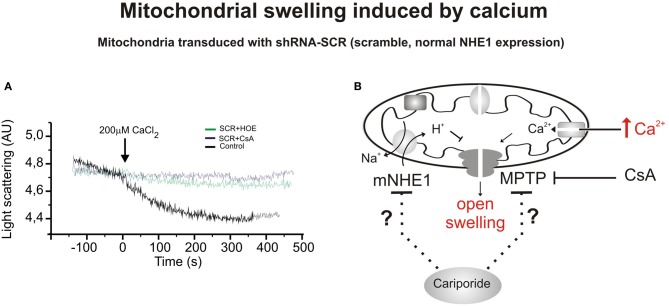
**Mitochondrial swelling induced by CaCl_2_. (A)** Typical experiment showing the scattered light absorbance traces of Ca^2+^-induced swelling in response to addition of CaCl_2_ to heart mitochondria isolated from rats transduced with shRNA-SCR (SCR, scrambled, control) in the presence or absence of cariporide (HOE642, 10 μ M) and Cyclosporine A (CsA, 10 mM). Cariporide and Cyclosporine attenuated Ca^2+^-induced mitochondrial swelling and the decrease in light scattering in mitochondrial suspensions. Cariporide inhibited the decrease in light scattering in a similar magnitude to CsA. **(B)** Scheme of a mitochondrion showing the CaCl_2_-induced swelling in mitochondria that expresses scrambled sequence and functional NHE1, and possible mitochondrial site of action of the cariporide. This inhibitor could act on different mitochondrial mechanisms, including NHE. They could act through a decrease in mitochondrial Ca^2+^, H^+^, and ΔΨ m affecting the MPTP formation or altering the sensitivity to those factors to induce MPTP formation. Modified from Villa-Abrille et al. (2011).

**Figure 2:**
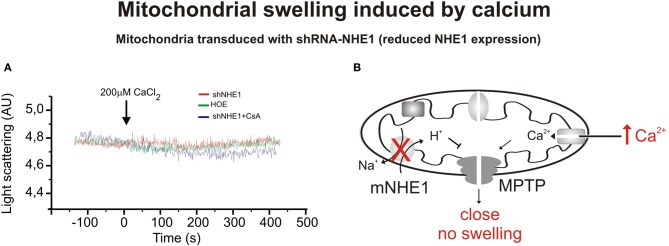
**Mitochondrial swelling induced by CaCl_2_. (A)** Typical experiment showing the scattered light absorbance traces of Ca^2+^-induced swelling in response to addition of CaCl_2_ for heart mitochondria isolated from rats transduced with shRNA-NHE1 in the presence and absence of cariporide. The shRNA-NHE1 transduction significantly inhibited Ca^2+^-induced mitochondrial swelling compared with SCR-transduced animals, (as previously shown in Figure 1). Cariporide and Cyclosporine attenuated the decrease in light scattering in SCR-transduced rats and did not affect the decrease in light scattering in shRNA-NHE1-transduced rats. These results indicate that the NHE1 participates in the mechanism by which the Ca^2+^ induces MPTP opening and swelling. **(B)** Scheme showing the absence of mitochondrial swelling and MPTP opening induced by Ca^2+^ when NHE1 is silencing. Modified from Villa-Abrille et al. (2011).

The related myocardial protective effects induced by CsA and NHE1 inhibition prompted us to speculate about the possibility that NHE1 inhibition protects the heart by targeting the mitochondria (Robin et al., 2007). We recently demonstrated that reduction of mNHE1 protein expression (shRNA-NHE1) or specific inhibition of NHE1 (HOE642) at the rat heart mitochondria significantly reduced the Ca^2+^-induced mitochondrial swelling [Figures 1, 2, modified from Villa-Abrille et al. (2011)]. Interestingly, HOE642 did not provide additional effect on Ca^2+^-induced mitochondrial swelling, in mitochondria with diminished levels of mNHE1 protein expression, suggesting a direct involvement of mNHE1 on the susceptibility of the MPTP for opening following the Ca^2+^ overload of the mitochondria. Additionally, the immunosuppressive agent, CsA inhibited the MPTP opening and mitochondrial swelling to the same extent as observed in isolated rat heart mitochondria inhibited with HOE642 or isolated mitochondria with reduced NHE1 expression [Figures 1, 2, modified from Villa-Abrille et al. (2011)]. We speculate that a decrease in mitochondrial exchanger function (or reduced expression by siRNA) should increase H^+^ concentration on the external side of the MPTP. In this regard, a prevention of MPTP formation by acidosis after an ischemic episode has been reported. Thus, accumulation of H^+^ following mNHE1 inhibition or expression reduction should decrease MPTP opening in heart mitochondria subjected to stress conditions. Though numerous studies (Karmazyn, 1988; Kusumoto et al., 2001; Wang et al., 2003; Fantinelli et al., 2006) demonstrated a protective role of NHE1 inhibition I/R injury in the heart, no mitochondrial effect of NHE1 inhibition has been considered. However, a report by Ruiz-Meana et al. (2003) drew attention to this possibility. Furthermore, Aldakkak et al. (2008) demonstrated that the enhanced activation of NHE with alkaline pH_i_ during ischemic stress leads to an additional increase in mitochondrial Ca^2+^ load, which contributes to greater deterioration of mitochondrial bioenergetics and ROS production on reperfusion. Authors proved that NHE1 blockade at an alkaline pH improved the functional recovery after reperfusion, minimized the increase in mitochondrial [Ca^2+^], preserved the mitochondrial redox state, and reduced ROS production. These data suggested that both sarcolemmal and mitochondrial NHE1 may be involved in promoting mitochondrial Ca^2+^ loading with I/R injury. In connection with this, Teshima and collaborators (2003) demonstrated that cariporide protects cardiomyocyte against cell death induced by oxidative stress, preserving intracellular Na^+^ and Ca^2+^ homeostasis and mitochondrial integrity. The mitochondrial Ca^2+^ overload and the mitochondrial ΔΨ m loss induced by oxidative stress were remarkably prevented by cariporide (Teshima et al., 2003). Additionally, Toda et al. (2007) demonstrated that the NHE1 inhibitor, cariporide, diminished the mitochondrial Ca^2+^ overload and MPTP opening induced by Na^+^/K^+^ ATPase inhibition in isolated cardiomyocytes. Although these effects were proposed to be secondary to prevention of the cytosolic increase in Ca^2+^, more recently a direct mitochondrial action of NHE1 inhibitors related to the suppression of the myocardial superoxide production has been reported (Garciarena et al., 2008). A “cyclosporine like effect” of NHE1 inhibition was proposed, but the site of action of those compounds was not identified (Garciarena et al., 2008). Garciarena et al. (2008) studied the “anti-ROS effect” of NHE1 inhibitors. The authors analyzed NADPH oxidase-dependent mitochondrial O_2_-generation induced by ANG II or ET-1. The NADPH oxidase-dependent mitochondrial release of ROS is the basis of the so-called “ROS-induced ROS release” phenomenon proposed by Zorov et al. (2000) and Kimura et al. (2005). However, there is no clear evidence that sarcolemmal NADPH oxidase-derived ROS interacts with the mitochondria. Accordingly, Zhang et al. (2001) using reconstituted mKATP channels of bovine heart demonstrated that O_2_-directly stimulates the opening of these channels. The three NHE1 inhibitors used by Garciarena et al. (2008) blunted the increased mitochondrial ROS production and the redox activation of the kinases, well-known downstream targets of ROS (Sabri et al., 1998; Rothstein et al., 2002). The authors proposed a direct mitochondrial effect rather than a scavenging action of NHE1 inhibitors. Additionally, they showed that cariporide blunted not only the increased O_2_-production induced by ANG II/ET-1 but also the production induced by opening the mKATP channel with diazoxide. A mitochondrial action of NHE1 inhibition secondary to changes in cytosol was previously suggested by Javadov et al. (2005). In two previous publications, these authors concluded that the mitochondrial effect of NHE1 inhibitors is indirect and possibly mediated by the prevention of cytosolic Ca^2+^ overload. Thus, inhibition of the sarcolemmal NHE1 alters intracellular Na^+^ concentration and promotes Ca^2+^ overload (Javadov et al., 2005, 2008). Accordingly, they did not detect any mitochondria direct effect of the NHE1 inhibitors, and they suggested an action on MPTP function through glycogen synthase kinase 3-β (Javadov et al., 2009). We were unable to determine whether or not under their experimental conditions the effect of NHE1 inhibitors on mitochondrial Ca^2+^ and/or H^+^ is prevented.

In 1969, Mitchell and Moyle (1969) proposed the existence of Na^+^/H^+^ exchange in mitochondria. Later, the existence of two systems capable of catalyze the H^+^ transport across the mitochondrial membrane, was described (Garlid et al., 1991). These systems—the unselective K^+^/H^+^ exchanger (KHE), which catalyzes the transport of virtually all alkali ions in the mitochondrion, and the NHE, which selectively transports Na^+^ or Li^+^ through mitochondrial membranes—were proposed as the main cation transporters of the mitochondria (Garlid et al., 1991). In addition, Garlid et al. (1991) demonstrated in beef heart mitochondria that solubilized NHE in the organelle can be purified in an active state.

In mitochondria, NHE mediates the exchange of matrix Na^+^ for intermembrane H^+^ generated by respiration (Garlid, 1988). Thus, H^+^ influx in the mitochondrial matrix through the mitochondrial NHE would constitute a form of H^+^ leakage not coupled to ATP synthesis, which in turn will dissipate the energy stored as transmitochondrial membrane H^+^ gradient.

NHE1 is a typical integral membrane protein with 10–12 predicted spanning segments, a long COOH terminus, and an NH2-terminal tail that possesses the mitochondrial localization signal. Previous studies using immunofluorescence and three-dimensional confocal microscopy techniques demonstrated the presence of NHE1 in the nuclear membranes isolated from the aortic vascular smooth muscle and liver of human, rabbit, and rat, suggesting a possible role of the nuclear NHE1 in the modulation of intranuclear pH (Bkaily et al., 2004). Our experiments, using isolated cardiomyocytes or mitochondrial lysates demonstrated the expression of the NHE1 protein in mitochondria isolated from rat ventricular myocardium and mitochondria isolated from HEK 293 cells transfected with NHE1-HA cDNA (Villa-Abrille et al., 2011). Besides the expression of NHE1 in the plasma membrane of cardiac cells, we have characterized the expression of NHE1 in mitochondria of cardiac muscle (Villa-Abrille et al., 2011) by several different experimental techniques (immunogold analysis combined with electron microscopy, immunohistochemistry combined with confocal microscopy or immunoblot analysis). Accordingly, Javadov et al. (2011) examined the expression of NHE1 and NHE6 in Percoll-purified rat heart mitochondria by immunoblot analysis and they only observed NHE1 in the mitochondria fraction (Javadov et al., 2011). Retention of NHE1 expression in isolated mitochondria subjected to digitonin allows them to conclude that NHE1 is expressed in the inner mitochondrial membrane (Javadov et al., 2011).

Dual distribution, at both plasma membrane and mitochondria, of others protein like the Na^+^/Ca^+2^ exchanger 1-3 (NCX1-3) (Gobbi et al., 2007), and Kv1.3 (Szabo et al., 2005), Kir6.2 (Garg and Hu, 2007), and Ca^+2^-activated BK potassium channels (Siemen et al., 1999), has been previously reported. In addition, Connexin 43 (Cx43), a constitutive protein that forms cardiac gap junctions contributing to cell-cell coupling, was also localized in mitochondria (Boengler et al., 2005, 2009). Cx43 contains four transmembrane domains as well as amino and carboxy termini located in the cytosol. Cx43 localized in subsarcolemmal mitochondria and its carboxy terminus directed toward the intermembrane space (Boengler et al., 2009).

With the assistance of the RNA interference technique, we evaluated the function and expression of NHE1 in cardiac tissues. With the objective of knocking down the expression of NHE1 in the heart, we injected a lentiviral vector expressing shRNA-NHE1 intramyocardically. The intramyocardial injection of lentivirus carrying a shRNA-NHE1 (silencing of NHE1) not only reduced the expression of NHE1 at the level of mitochondria but also prevented Ca^2+^-induced swelling of rat heart mitochondria [Figures 1, 2, modified from Villa-Abrille et al. (2011)]. The silencing of NHE also prevents the development of the slow force response (Perez et al., 2011). A decrease in mitochondrial NHE function (or reduced expression by siRNA) should increase H^+^ concentration in mitochondrial matrix. In connection with this, the prevention of MPTP formation by acidosis in reperfusion after ischemia has been reported (Cohen et al., 2007; Rodriguez-Sinovas et al., 2009). We reported a delay of MPTP formation in isolated mitochondria by NHE1 inhibition and posttranscriptional NHE1 gene silencing. These findings open a new avenue of research about the mechanism of protection achieved by NHE1 inhibition in reperfusion injury, cardiac hypertrophy, and heart failure. The mechanism by which the opening of the MPTP is prevented at low NHE1 expression is unknown. As previously described under physiological conditions, the mitochondrial NHE introduces cytosolic H^+^ into the mitochondrial matrix in exchange for mitochondrial Na^+^. Therefore, a decrease in exchanger activity or expression should reduce H^+^ and increase Na^+^ concentration in the mitochondrial matrix and perhaps increase H^+^ concentration on the cytosolic side of the MPTP. The decrease in H^+^ matrix concentration would favor and not prevent MPTP formation, but the increase of H^+^ on the cytosolic side of the MPTP may inhibit pore formation. In connection with this scenario, the prevention of MPTP formation by acidosis in reperfusion after ischemia has been reported (Cohen et al., 2007). An increase in mitochondrial Na^+^ would decrease the inwardly directed Na^+^ gradient, affecting the mitochondrial Na^+^/Ca^2+^ exchanger, other factors being constant (Murphy and Steenbergen, 2008). A decrease in Ca^2+^ efflux from the mitochondria would increase mitochondrial Ca^2+^ concentration and favor rather than reduce MPTP formation.

The main objective of this review was to emphasize the presence of NHE1 in the mitochondrial membrane and its role in MPTP opening. We are proposing the mitochondrial NHE1 as a novel target to prevent cardiac disease including I/R injury, cardiac hypertrophy, and heart failure. However, we could not rule out the concomitant participation of the sarcolemmal NHE1 in the protective effects achieved by NHE inhibition.

### Conflict of interest statement

The authors declare that the research was conducted in the absence of any commercial or financial relationships that could be construed as a potential conflict of interest.
